# Chlorhexidine in cosmetic products a market survey

**DOI:** 10.1186/2045-7022-4-S3-P69

**Published:** 2014-07-18

**Authors:** Morten Schjørring Opstrup, Jeanne Duus Johansen, Rossana Bossi, Michael Dyrgaard Lundov, Lene Heise Garvey

**Affiliations:** 1 Copenhagen University Hospital Gentofte, National Allergy Research Centre, Denmark; 2 Aarhus University, Institut for Bioscience - Arctic Research Centre, Denmark; 3 Copenhagen University Hospital Gentofte, Danish Anaesthesia Allergy Centre, Allergy Clinic, Denmark

## Background

Chlorhexidine may cause both type I and type IV allergy, but the mode of sensitization is unknown. It is a widely used disinfectant in the health care setting as well as an ingredient in cosmetic products, but the extent of use is unknown. According to the European Cosmetic Directive, chlorhexidine is allowed in cosmetic products in a concentration of up to 0.3%. The aim of this study was to identify which types of cosmetic products that contain chlorhexidine, and to measure their concentration of chlorhexidine.

## Method

The study took place February 2013 April 2013 in Copenhagen, Denmark. All cosmetic products in 14 supermarkets, one hairdresser, one beauty and retail store and one optician were checked for chlorhexidine by reading the ingredient label. All products were photographed and product names noted to avoid duplicates. The products containing chlorhexidine were purchased. Chlorhexidine concentration was measured in ten selected products by high-performance liquid chromatography (HPLC) with ultraviolet (UV)-detector.

## Results

Chlorhexidine was found in 82 out of 2310 cosmetic products (3.5%) in the following categories: Conditioners (n=30), hair dyes (n=13), hair treatments (n=10), creams (n=9), face washes (n=4), wet wipes (n=4), hair styling products (n=4), skin tonics (n=3), contact lens fluids (n=2), make up removers (n=2) and mouth washes (n=1). The concentration of chlorhexidine was measured in ten products to 0.03% 1.54%. Two products, both creams, had a concentration above the allowed 0.3% (0.43% and 1.54%, respectively).

## Conclusion

In this market survey, chlorhexidine was identified in 82 different cosmetic products (3.5%), predominantly hair products. Two creams had a concentration of chlorhexidine higher than allowed by the European Cosmetic Directive. The extent of use of chlorhexidine in the health care setting as well as the relevance for allergic sensitization should be further explored.

